# The pharmacokinetics of cefazolin in patients undergoing elective & semi-elective abdominal aortic aneurysm open repair surgery

**DOI:** 10.1186/1471-2253-11-5

**Published:** 2011-02-22

**Authors:** Alexandra Douglas, Mahdi Altukroni, Andrew A Udy, Michael S Roberts, Kersi Taraporewalla, Jason Jenkins, Jeffrey Lipman, Jason A Roberts

**Affiliations:** 1 Burns, Trauma and Critical Care Research Centre, School of Medicine, The University of Queensland, Brisbane, Queensland, Australia; 2 Department of Intensive Care Medicine, Royal Brisbane and Women's Hospital, Queensland, Australia; 3 Therapeutics Research Unit, The University of Queensland, Brisbane, Australia; 4 School of Pharmacy, University of South Australia, Adelaide, Australia; 5 Department of Anaesthesia, Royal Brisbane and Women's Hospital, Queensland, Australia; 6 Department of Vascular Surgery, Royal Brisbane and Women's Hospital, Queensland, Australia; 7 Pharmacy Department, Royal Brisbane and Women's Hospital, Queensland, Australia

## Abstract

**Background:**

Surgical site infections are common, so effective antibiotic concentrations at the sites of infection are required. Surgery can lead to physiological changes influencing the pharmacokinetics of antibiotics. The aim of the study is to evaluate contemporary peri-operative prophylactic dosing of cefazolin by determining plasma and subcutaneous interstitial fluid concentrations in patients undergoing elective of semi-elective abdominal aortic aneurysm (AAA) open repair surgery.

**Methods/Design:**

This is an observational pharmacokinetic study of patients undergoing AAA open repair surgery at the Royal Brisbane and Women's Hospital. All patients will be administered 2-g cefazolin by intravenous injection within 30-minutes of the procedure. Participants will have samples from blood and urine, collected at different intervals. Patients will also have a microdialysis catheter inserted into subcutaneous tissue to measure interstitial fluid penetration by cefazolin. Participants will be administered indocyanine green and sodium bromide as well as have cardiac output monitoring performed and tetrapolar bioimpedance to determine physiological changes occurring during surgery. Analysis of samples will be performed using validated liquid chromatography tandem mass-spectrometry. Pharmacokinetic analysis will be performed using non-linear mixed effects modeling to determine individual and population pharmacokinetic parameters and the effect of peri-operative physiological changes on cefazolin disposition.

**Discussion:**

The study will describe cefazolin levels in plasma and the interstitial fluid of tissues during AAA open repair surgery. The effect of physiological changes to the patient mediated by surgery will also be determined. The results of this study will guide clinicians and pharmacists to effectively dose cefazolin in order to maximize the concentration of antibiotics in the tissues which are the most common site of surgical site infections.

## Background

Surgical site infections are of significant concern to the healthcare system and are the second most common type of adverse-event occurring in hospitalized patients [1]. Patients undergoing clean-contaminated and clean surgeries are reported to have infection rates of 11% and 1% respectively [2]. The consequences of such complications are dependent on the sites of infections as well as disease severity. Additionally, patients will be exposed to significant morbidities and mortalities including bronchopneumonia (8%), renal insufficiency (5%) and wound infection (3%) as well as myocardial infarction (3%), stroke (2%) and pulmonary embolism (1%) respectively [3]. They will also suffer the financial burden of a prolonged hospital stay, thereby also impacting on the health care system. An attempt to minimise such complications will provide significant cost-effectiveness advantages for the health care system. Furthermore, the inappropriate use of antibiotics will impose selective antibiotic pressure in the hospital environment. Antibiotics are considered an essential part of the surgical prophylaxis strategy. Identifying the pharmacokinetic properties of antibiotics and the likely alteration during surgical interventions would facilitate rational prescription.

Different types of surgeries will have different levels of effect on pharmacokinetics. For example, burn patients undergoing excisions and bone grafting can have dramatically different antibiotic pharmacokinetic parameters compared with healthy volunteers. In a previous study from our group we were able to show that conventional single peri-operative antibiotic prophylaxis did not achieve adequate plasma concentrations above established minimum inhibitory concentrations (MIC) for target organisms during lengthy debridement procedures [4]. Antibiotic concentrations in the interstitial fluid of tissues are likely to be more informative than plasma concentrations in this setting, as tissues are far more likely to be the site of infection [5].

Another common surgical procedure for which little data is available to describe the interstitial fluid concentrations of prophylactic antibiotics is abdominal aortic aneurysm (AAA) open repair surgery. This type of surgery often results in blood losses in large quantities requiring the need for volume replacement as well as vasopressors and inotropic support. Each of these factors is capable of increasing the clearance of hydrophilic compounds such as the antibiotics typically used for prophylaxis in this population [6]. Other confounding factors in this population include the age of these patients (mostly 65-75 years) and the fact that many patients have complications of diabetes and hypertension including nephropathy and vasculopathy [3,7]. In addition, these patients are going to have major blood vessels surgically clamped temporarily reducing kidney perfusion and potentially altered perfusion of other tissues. Consequently, tissue concentrations of antibiotics may be inadequate. In light of this, it appears that there are many complexities in achieving adequate tissue concentrations of antibiotics. The complex interrelationship of altered physiology and pharmacokinetics has been recently described [6,8].

The relationship between surgical site infections and the sub-optimal antibiotic concentrations during surgery has been established for aminoglycosides but not specifically for beta-lactams [9]. Therefore, obtaining the concentrations of antibiotics at the sites of infection will be beneficial to enhance our understanding of the pharmacokinetics of our selected antibiotics. Tissue concentrations of antibiotics are best determined by a technique known as microdialysis [10,11].

Cefazolin is recommended by the Australian Therapeutic Guidelines for prophylaxis in vascular surgery. It belongs to the cephalosporin class of antibiotics and provides an excellent coverage against Gram positive bacteria and limited activity against Gram negatives. Cephalosporins display time-dependent killing meaning maximal bacterial killing requires maximal duration of exposure with the time for which the unbound (or free) antibiotic concentration is maintained above the MIC (*f *T_> MIC_) being the index correlated with efficacy. To the best of our knowledge, there are no studies that describe tissue concentrations of antibiotics during AAA open repair surgery. We hypothesise that measurement of these concentrations will enable procurement of doses that could optimize the concentrations of antibiotics in the interstitial space, where infections are likely to occur.

### Aims

To describe the pharmacokinetics of cefazolin administered intravenously to patients undergoing elective and semi-elective AAA open repair surgery: to subsequently develop a rational physiological population pharmacokinetic model that describes plasma and tissue concentrations of cefazolin in these patients.

## Methods/Design

This is an observational pharmacokinetic study. Blood, urine, and microdialysate samples will be collected at established intervals from participants presenting to the Royal Brisbane and Women's Hospital, undergoing elective or semi-elective AAA open repair surgery.

### Setting

The Intensive Care Unit at the Royal Brisbane and Women's Hospital is a 30-bed tertiary referral unit with 2000-2500 admissions per year. This study is done in collaboration with the Burns, Trauma and Critical Care Research Centre, School of Medicine, the University of Queensland, as well as the Departments of Anaesthesia and Vascular Surgery at the Royal Brisbane and Women's Hospital.

### Identification of eligible patients

Patients undergoing elective and semi-elective AAA open repair surgery, who fulfil the inclusion and exclusion criteria will be eligible for the study. Written informed consent will be obtained from each participant.

#### Inclusion Criteria

1. Age > 18 years and < 90 years.

2. Patients scheduled for elective and semi-elective Abdominal Aortic Aneurysm (AAA) repair.

3. Prescription of intravenous cefazolin for peri-operative surgical prophylaxis.

4. An arterial line in situ.

5. Informed consent by the patient or a legally authorized representative to participate in the study and to store specimens for immediate and future analysis.

#### Exclusion criteria

1. Prior prescription of cefazolin within the preceding 72-hours.

2. Known allergy to the study drug.

3. Patients whose religion or personal beliefs limit the physician's ability to conduct standard resuscitation during surgery (e.g. Jehovah's Witness).

### Participants

Participants will be identified by either a Consultant Anaesthetist or a Vascular Surgeon. All participants will receive the same dose of cefazolin and will receive the same protocol.

### Drug dosing

All participants will receive cefazolin 2 g (Cephazolin, DBL, Sydney Australia) in 10 ml 0.9% sodium chloride as a 3-minute slow bolus intravenous injection within 30-minutes of the first incision of surgery.

Patients will be given marker compounds to determine any alteration in the patient's physiology as a result of the surgery. Indocyanine green (ICG) - Pulsion 0.5 mg/kg (maximum dose 50 mg) will be administered to provide a measure of plasma volume, an indication of hepatic function and the distribution kinetics of highly bound solutes. Plasma volume will be obtained from the last ICG concentration [12]. The ICG will be administered as a rapid bolus via central venous line 10 min prior to the start of the antibiotic infusion. ICG non-invasive oximetry measurements will be taken over a period of 7 min.

Sodium bromide (5% w/v solution; 1 ml/kg; Central Pharmacy, Brisbane, Australia) will be administered intravenously to measure extracellular and intracellular fluid fluctuations [13]. It will be administered as a 2.5 g slow bolus over 3 min. Sodium bromide will be administered starting 3 min before administration of cefazolin.

### Microdialysis

The principles and details of microdialysis have been described previously [10]. Briefly, microdialysis is based on the sampling of analytes from the extracellular space by diffusion across a semi-permeable membrane. In vivo, this process is accomplished by constantly perfusing the microdialysis probe with a physiological solution at a low flow rate. Once the probe is implanted in tissue, analytes diffuse across the membrane from the extracellular fluid into the perfusate and may be sampled and analysed. In this study, a microdialysis probe (CMA 60, Microdialysis AB, Stockholm, Sweden) with a molecular weight cut off of 20 kD, an outer diameter of 0.6 mm and a membrane length of 30 mm was aseptically placed in the subcutaneous tissue of the upper arm of each patient. The probe will be perfused with cefalotin (10 mg/L; internal standard; Cephalothin, DBL, Sydney Australia) in 0.9% sodium chloride at a flow rate of 1.6 μl/min [10]. The recovery of cefazolin in the microdialysate solution was interpolated from the loss of internal standard (cefalotin) across the microdialysis membrane into tissue according to the retrodialysis method [14,15].

$$\text{\% cefazolin recovery = 1}00\;\times \;\left({\text{C}}_{\text{in}}-{\text{mean C}}_{\text{out}}/{\text{C}}_{\text{in}}\right)$$

Where C_in _= cefalotin 2 mg/L (perfusate); C_out _= measured cefalotin concentration in microdialysate

### Other measurements performed

#### Tetrapolar Bioimpedance

An experienced clinician will perform tetrapolar bioimpedance on two occasions during the study. The first measurement will occur 20 minutes prior to the antibiotic dose and then repeated at the conclusion of sampling. The results will be used for measuring the fat free mass [11] of the patient and detecting altered fluid status over the study period and to calculate the drug volume of distribution.

#### Cardiac Output

Non-invasive pulse contour arterial waveforms will be taken to measure cardiac output and other derived measurements utilizing the FloTrac/Vigileo™ system (Edwards Lifesciences, Irvine, CA, USA). Obtained cardiac output results will be confirmed using an UltraSonic Cardiac Output Monitor (The USCOM ultrasonic cardiac output monitor USCOM Pty, Coffs Harbour, NSW, Australia). These measurements will be compared at three times points (30-minutes prior to antibiotic administration, 2 hours after antibiotic administration and then 60-minutes after skin closure).

### Sample collection

All participants will have blood sampling at the following times, 0 minutes (prior to the dose), 3, 10, 30, 60, 90, 180, 300, and finally at 480 (minutes) following the dose or until the end of surgery.

Urine will be collected at two-hourly intervals during pre- and peri-operative periods. A 10 ml aliquot will be used to determine renal clearance of cefazolin in the two-hour time interval with the balance of urine used to calculate 2-hour urinary creatinine clearance. Further to this, an eight-hourly urinary creatinine clearance will also be taken post-operatively to determine the participant's renal function at the end of the procedure.

Microdialysate samples will be collected at 30-minute intervals during the pre- and the post-operative periods to provide a continuous description of interstitial concentrations of cefazolin.

### Sample handling and storage

Blood will be centrifuged at 3000 rpm for 10 minutes before being stored at -80°C until assay.

### Sample analysis

Analysis of plasma, urine and microdialysis samples will be performed by validated liquid chromatography tandem-mass spectrometry (LC-MS/MS) methodology at the Therapeutics Research Unit, The University of Queensland.

### Data collection

Additional data will be obtained from the participant or extraction from the clinical notes. These include:

1. Participant information - age, gender, weight, height, allergies.

2. Clinical details including the admitting diagnosis, the progress, and the outcome.

3. Laboratory investigations - full blood count, electrolytes, liver function tests, arterial blood gasses, and coagulation profile.

4. Details of surgical procedure - aortic clamp timing and duration, fluid administration requirements.

5. Results of all microbiological studies (performed as a part of standard practice).

6. Glomerular filtration rate will be estimated from creatinine clearance but it will be calculated from serum creatinine and the urine sample collected at eight-hours post-operatively during the initial study period.

7. Co-morbidities.

8. Evidence of organ dysfunction.

### Statistical considerations

This is a non-interventional study and is not intended to compare between different treatment modalities and therefore a power calculation is not possible. The purpose of the study is to identify the significant covariates that can describe the variability in cefazolin pharmacokinetics in participants undergoing AAA repair surgery. A sample size of twelve is considered sufficient to identify the most clinically significant covariates in this pharmacokinetic study. In general, population pharmacokinetic studies have fewer statistical design restrictions [16].

### Data analysis

The results of the sample analysis will be analysed using a non-linear mixed effects modelling approach with the pharmacokinetic computer software program NONMEM^® ^(GlobMax LLC, Hanover, MD, USA) to develop a model for cefazolin dosing via the intravenous route when given as a prophylaxis during AAA open repair surgery. Pharmacokinetic parameters (volume of distribution, clearance, and half lives) will be compared with already published data using the Mann-Whitney test. The influence of demographics and clinical covariates will be evaluated as well. The model will simulate cefazolin pharmacokinetics for patients undergoing AAA open repair surgery to best evaluate the adequacy of a single dose of 2 g of cefazolin, intravenously given as surgical prophylaxis.

### Ethical considerations

The Human Research and Ethics Committee of the Royal Brisbane and Women's Hospital (2007/187) and the medical Research Ethics committee of The University of Queensland (2008002032) have approved this study.

### Withdrawal from the study

Participants may withdraw from the study at anytime without prejudice, as documented and explained at the time of consenting.

## Discussion

Surgical site infections can be minimized by optimizing peri-operative antibiotic prophylaxis doses. The current study is the first to document plasma and interstitial fluid levels of cefazolin in patients undergoing AAA open repair surgery. Further, we are unaware of any other studies that describe the changing physiology of the patient during the peri-operative period in the proposed level of detail. We believe that maximizing the concentration of antibiotics at the likely sites of infections, namely the interstitial fluid, will help to obviate the problem of inadequate prophylaxis. Patients undergoing AAA repair surgery are expected to undergo physiological changes related to fluid and vasopressor administration and aortic clamping likely to affect antibiotic volumes of distributions and clearance. In light of this, these patients may be at risk of inadequate antibiotic concentrations in the interstitial fluid of tissues.

In conclusion, our study will describe the likely adequacy of cefazolin when administered intravenously as a prophylaxis to patients undergoing AAA open repair surgery by measuring its concentration at tissues using microdialysis [10,11]. Various peri-operative pathophysiological changes will also be measured in an attempt to understand antibiotic distribution variables.

## Abbreviations

AAA: Abdominal Aortic Aneurysm; MIC: Minimal inhibitory concentration; *f *T_> MIC_: time for which the unbound (or free) antibiotic concentration is maintained above the MIC; ICG: indocyanine green; C_in_: microdialysis perfusate concentration; C_out_: microdialysis dialysate concentration; USCOM: UltraSonic Cardiac Output Monitor; NONMEM: Non-linear Mixed effect modeling;

## Competing interests

The authors declare that they have no competing interests.

## Authors' contributions

AD, MA, MSR, JL, JAR designed the study and wrote the protocol. KT and JJ provided input and advice and will be involved throughout the study. AAU provided input and advice and will be involved with recruitment component of the study. All authors read and approved the final manuscript.

## Pre-publication history

The pre-publication history for this paper can be accessed here:

http://www.biomedcentral.com/1471-2253/11/5/prepub
